# How is 3D modeling in metabolic surgery utilized and what is its clinical benefit: a systematic review and meta-analysis

**DOI:** 10.1097/JS9.0000000000002301

**Published:** 2025-02-26

**Authors:** Henry Douglas Robb, Aksaan Arif, Rithvik Mahadev Narendranath, Bibek Das, Khaled Alyaqout, William Lynn, Yasser Abul Aal, Hutan Ashrafian, Matyas Fehervari

**Affiliations:** a Department of Surgery and Cancer, Imperial College London, London UK; b Jaber Al Ahmad Hospital, Kuwait City Kuwait; c Gastrointestinal Surgery, Maidstone and Tunbridge Wells NHS Trust, Tunbridge Wells, London UK

**Keywords:** 3D printing, augmented reality, computer-generated 3D imaging, gastrointestinal disease, general surgery, metabolic surgery, virtual reality

## Abstract

**Background:**

Three-dimensional (3D) modeling is an emerging technology in surgery, with applications in operative planning, surgical education, and patient engagement. Metabolic surgery, the most effective treatment for obesity, is increasingly prevalent leading to new complex clinical challenges. This systematic review aims to understand the use of 3D modeling in metabolic surgery and its impact on clinical outcomes.

**Methods:**

Following a registered protocol (PROSPERO: CRD42024545311), a comprehensive search using MEDLINE, Embase, and CENTRAL Cochrane Library was conducted. Eligible papers underwent screening and full-text review. A qualitative thematic analysis was performed alongside meta-analyses on available volumetric data. Results were reported as directed by the PRISMA guidelines.

**Results:**

Twenty-nine studies were included, with most at Level II evidence (*n* = 19, 66%). Studies focused on operative planning and surgical practice (90%, *n* = 26) and were subdivided into preoperative planning (14%, *n* = 4), postoperative diagnosis (31%, *n* = 9), and postoperative assessment and prediction (45%, *n* = 13). Only three papers addressed surgical education (10%). 3D modeling for patient education was unexplored. To assess 3D modeling’s cross-study consistency, pooled meta-analyses on preoperative and postoperative 3D gastric volumetry and abdominal circumference were performed. Average preoperative stomach volume was 794.93 mL (95% confidence interval [CI]: 518.61–1071.26 mL). Postoperative LSG and RYGB/OAGB gastric volumes were 171.71 mL (95% CI: 113.37–288.58 mL) and 35.73 mL (95% CI: 29.32–42.14 mL) respectively. Average abdominal circumference was 120.04 cm (95% CI: 100.72–139.35 cm). All volumes were consistent with published data.

**Conclusions:**

This systematic review highlighted the accuracy of 3D modeling for volumetric assessments and its developing role in surgical planning and training. However, its potential benefits in AR or 3DP models, in patient education or for answering bariatric surgical debates using 3D volumetric studies remain underutilized.

## Introduction

**Highlights**

- **Accuracy of 3D Modelling:** 3D modelling is highly accurate for volumetric assessments in metabolic surgery, particularly in the measurement of gastric volumes pre- and post-operatively.
- **Operative Planning and Diagnosis:** 3D modelling is predominantly used in operative planning and surgical practice. It is especially beneficial for pre-operative guidance, diagnosing post-operative complications, and assessing post-operative anatomy.
- **Impact on Surgical Training:** While 3D modelling is making inroads into surgical training, particularly with virtual reality (VR) applications, its full potential in surgical education and certification processes is yet to be fully realized.
- **Underutilized Potential:** The review highlights missed opportunities in further exploring the role of 3D modelling, particularly in patient education.
- **Challenges and Future Directions:** Significant heterogeneity in outcome measures and a lack of standardization in reporting pose challenges to fully understanding the clinical benefits of 3D modelling. Future research is needed to standardize volumetric assessments and associated clinical outcomes.

As medical imaging technology has rapidly advanced^[^1,2^]^, three-dimensional (3D) modeling has emerged as a powerful innovation. 3D modeling describes the creation of either virtual reality (VR), augmented reality (AR), or 3D-printed (3DP) reconstructions of patient anatomy and pathology^[^3,4^]^. The applications of 3D modeling have been broad, from pediatrics to oncological surgery^[^5,6^]^. For surgeons, 3D modeling has become particularly beneficial with growing roles in surgical training, patient education, and surgical practice.

With one in eight people suffering with obesity and its multi-morbid complications^[^7^]^, it is unsurprising rates of metabolic surgery have risen 10-fold over the last 20 years^[^8^]^. While metabolic surgery is the most effective treatment for obesity^[^9^]^, it has introduced new clinical challenges for physicians. For example, procedures such as one-anastomosis gastric bypass (OAGB), single anastomosis duodenal-ileal bypass with sleeve, and duodenal switch are becoming increasingly common, and general surgeons are likely to encounter these varied anatomies. These operations can increase the risk of complications such as internal hernia, which may be challenging to diagnose using conventional imaging. Furthermore, the complexities of bariatric surgery can be technically difficult for training surgeons and simultaneously demanding for patients to understand^[^10,11^]^.

Reassuringly, 3D modeling is a promising solution to overcome these challenges. Physical and virtual reconstruction has been shown to aid diagnosis^[^12^]^, improve surgical training^[^13^]^, and offer substantial opportunities for patient education^[^14^]^. For example, the use of 3D computed tomography imaging can enhance the detection and assessment of internal hernias, providing more detailed anatomical visualization and improved diagnostic accuracy^[^15^]^. Therefore, the authors undertook a systematic review and meta-analysis aiming to identify the current clinical applications of 3D modeling within metabolic surgery and assess their clinical benefits.

## Methods

### Protocol and registration

An *a priori* systematic review protocol was developed according to internationally accepted guidelines, with findings reported in line with the Preferred Reporting Items for Systematic Reviews and Meta-Analyses and Assessing the methodological quality of systematic reviews guidelines^[^16,17^]^ (Supplemental digital contents 1 and 2, available at: http://links.lww.com/JS9/D979; http://links.lww.com/JS9/D980). The review protocol was publicly registered and can be accessed on the PROSPERO Centre for Reviews and Dissemination (CRD42024545311).

The systematic review and meta-analysis aimed to be the first review to establish the utilization of 3D modeling within metabolic surgery and perform meta-analyses on clinically relevant outcomes. As the applications of 3D modeling are expected to be disparate, an initial narrative summary of included papers will be performed.

### Eligibility criteria

To be considered for inclusion, identified literature must:

1. Be original research.
2. Use 3D Modelling to reconstruct gastrointestinal (GI) anatomy.
3. Focus on metabolic or bariatric surgery.
4. Be randomized controlled trials (RCT), prospective or retrospective cohort studies, case (control) studies, cross-sectional studies, or case study/series.

Non-human research studies, papers not published in peer-reviewed literature, studies not written in English and 3D modeling outwith the context of metabolic surgery were excluded. 3D modeling is an emerging technology, especially in the field of metabolic surgery. Therefore, the authorship team expected a relatively high proportion of low-level evidence^[^18^]^, and decided not to exclude studies based on a quality assessment to prevent limiting the breadth of the review.

### Information sources and search strategy

A comprehensive search strategy was created by the lead investigator collaborating with a specialist medical librarian. After initial pilot searches, tailored search strategies using keywords, thesauri terms (MeSH terms (MEDLINE and EMTREE (Embase)) and Boolean operators were created for MEDLINE, Embase, and CENTRAL Cochrane Library (Supplemental Digital Content 3, available at: http://links.lww.com/JS9/D981). Databases were searched from their inception to April 2024. Grey literature searches were performed using OpenGrey and Grey Literature Report. Identified literature was collated using EndNote V.X9 (Clarivate) to optimize duplicate removal prior to transfer to Covidence, a web-based software platform for systematic literature reviews supported by the Cochrane Collaboration^[^19^]^.

### Selection process

A two-stage screening process “title and abstract screening” and “full-text review” was performed by two independent reviewers on identified studies for topic relevance. Any disagreement was resolved through discussion, and if required, a third reviewer provided the decisive vote. Satisfactory inter-rater agreement was achieved with a moderate Cohen’s kappa of 0.58^[^20^]^.

### Data collection, data items, and risk of bias

To accommodate the diverse portfolio of included studies, a data extraction form was created with quantitative and qualitative thematic components to allow for both narrative and data-driven assessments. The data template included study descriptors (authorship, study design, patient number, imaging modality, 3D modeling technique, follow up length), thematic allocation (surgical education and training, patient education and engagement, and surgical planning and procedure) and clinical endpoints (such as excess weight loss, total weight loss [TWL] and postoperative stomach volume). Data extraction was performed independently by three authors. All identified literature were non-randomized, therefore the Newcastle–Ottawa Scale (NOS) was used to grade risk of bias and completed by two independent reviewers^[^21^]^.

### Certainty of evidence

To assess certainty of evidence, the Grading of Recommendations Assessment, Development and Evaluation (GRADE) framework was applied^[^22^]^. In brief, evidence based on RCTs is initially classed as high certainty and those founded in observational studies as low, using a scale of very low, low, moderate and high certainty. Evidence is either upgraded or downgraded based on five components: risk of bias, inconsistency, imprecision, indirectness, and publication bias^[^23^]^.

### Statistical analysis

Statistical analysis was performed using Stata Software, Version 15.1. StataCorp LCC, TX. Random effects analysis was used to calculate weighted mean differences and mass effect. All studies were included in the meta-analysis if relevant data were available. Data were pooled using a random effects model and statistical heterogeneity was calculated using *I*^2^. As per Cochrane Collaboration guidance, an *I*^2^ of <30% was considered minimal heterogeneity, between 30% and 60% to be moderate heterogeneity, and >60% was substantial heterogeneity^[^24^]^.

## Results

### Study selection

**Figure 1.**
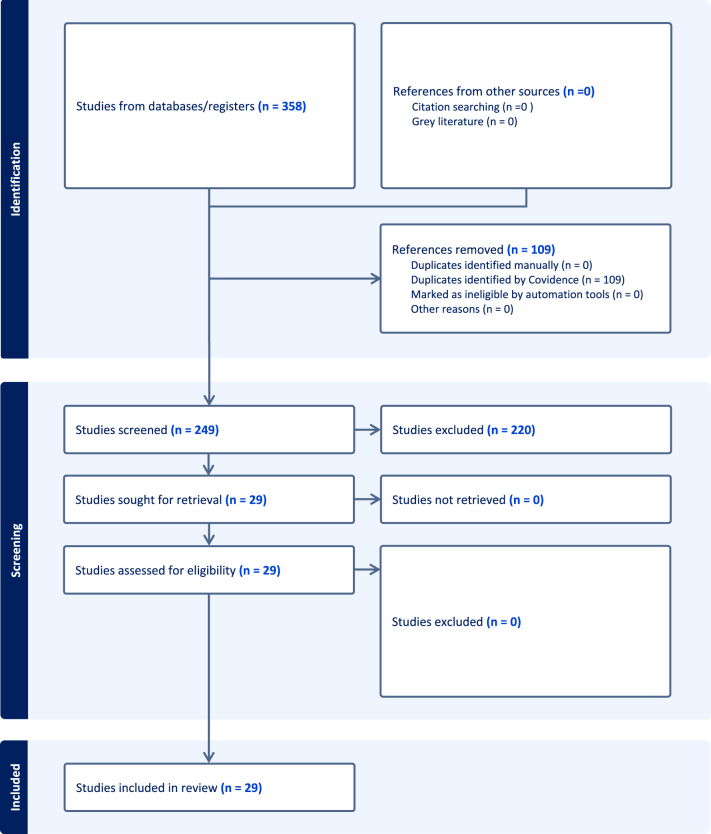
PRISMA flowchart

In total, the search identified 358 papers. After duplicate removal, 249 studies underwent screening and full-text review. Ultimately, 29 papers were included for extraction and data analysis (Fig. 1). The most frequent reason for exclusion at full-text review was either failure to publish in peer-review literature or 3D modeling outside GI tract.

### Study characteristics

As expected, as an emerging technology, most papers (66%, *n* = 19) were published within the last 5 years and the earliest publication was 2008 (Table 1) ^[^25–53^]^. The majority of identified literature originated in Europe (52%, *n* = 15). Study designs were predominantly prospective (66%, *n* = 19) or retrospective (31%, *n* = 9) cohorts. A single case report was included. There were no randomized control trials. Overall, the level of evidence was relatively low with 66% (*n* = 19) Level II and remaining work Level III or below. All studies created virtual models, with no 3DP or AR applications. Segmentation method was poorly reported, however when stated most studies used automated approaches (31%, *n* = 9).

**Table 1:** Study characteristics

Author, year	Origin	Design	Theme	Sub-theme	Sample size
Kim *et al* 2020	Republic of Korea	Prospective cohort study	Operative planning and surgical practice	Preoperative planning	100
Toniolo *et al* 2022	Italy	Prospective cohort study	Operative planning and surgical practice	Preoperative planning	23
Debs *et al* 2020	France	Case report	Operative planning and surgical practice	Preoperative planning	1
Felsenreich *et al* 2023	Austria	Retrospective cohort study	Operative planning and surgical practice	Preoperative planning	50
Sabry *et al* 2022	Egypt	Retrospective cohort study	Operative planning and surgical practice	Postoperative diagnosis	15
Arnoldner *et al* 2020	Austria	Prospective cohort study	Operative planning and surgical practice	Postoperative diagnosis	30
Elredge *et al* 2020	Australia	Prospective cohort study	Operative planning and surgical practice	Postoperative diagnosis	18
Baumann *et al* 2011	Germany	Retrospective cohort study	Operative planning and surgical practice	Postoperative diagnosis	27
Wickremasinghe *et al* 2024	Australia	Prospective cohort study	Operative planning and surgical practice	Postoperative assessment and prediction	79
Chen *et al* 2024	Taiwan	Prospective cohort study	Operative planning and surgical practice	Postoperative assessment and prediction	63
Sahin *et al* 2023	Turkey	Prospective cohort study	Operative planning and surgical practice	Postoperative assessment and prediction	49
Riccioppo *et al* 2018	Brazil	Retrospective cohort study	Operative planning and surgical practice	Postoperative assessment and prediction	67
Robert *et al* 2016	France	Prospective cohort study	Operative planning and surgical practice	Postoperative assessment and prediction	67
Blanchet *et al* 2010	France	Retrospective cohort study	Operative planning and surgical practice	Postoperative diagnosis	20
Yamaguchi *et al* 2021	Japan	Retrospective cohort study	Operative planning and surgical practice	Postoperative assessment and prediction	40
Klop *et al* 2018	The Netherlands	Prospective cohort study	Operative planning and surgical practice	Postoperative diagnosis	15
Hanssen *et al* 2017	Venezuela	Prospective cohort study	Operative planning and surgical practice	Postoperative assessment and prediction	32
Mohsen Abd-Elfattah Moursi *et al* 2022	Egypt	Retrospective cohort study	Operative planning and surgical practice	Postoperative assessment and prediction	30
Alva *et al* 2008	USA	Retrospective cohort study	Operative planning and surgical practice	Postoperative assessment and prediction	3
Robert *et al* 2014	France	Prospective cohort study	Operative planning and surgical practice	Postoperative assessment and prediction	39
Karila-Cohen *et al* 2022	France	Prospective cohort study	Operative planning and surgical practice	Postoperative diagnosis	194
M. Felsenreich *et al* 2020	Austria	Prospective cohort study	Operative planning and surgical practice	Postoperative diagnosis	12
Lin *et al* 2020 A	Taiwan	Prospective cohort study	Operative planning and surgical practice	Postoperative assessment and prediction	32
Pawanindra *et al* 2014	India	Prospective cohort study	Operative planning and surgical practice	Postoperative assessment and prediction	22
Ayuso *et al* 2022	USA	Retrospective cohort study	Operative planning and surgical practice	Postoperative assessment and prediction	122
Disse *et al* 2016	France	Prospective cohort study	Operative planning and surgical practice	Postoperative diagnosis	54
Lewis *et al* 2012	England	Prospective cohort study	Surgical education	VR simulation	20
Giannotii *et al* 2023 A	Italy	Prospective cohort study	Surgical education	VR simulation	20
Barre *et al* 2019	France	Prospective cohort study	Surgical education	VR simulation	10

### Thematic analysis

**Figure 2.**
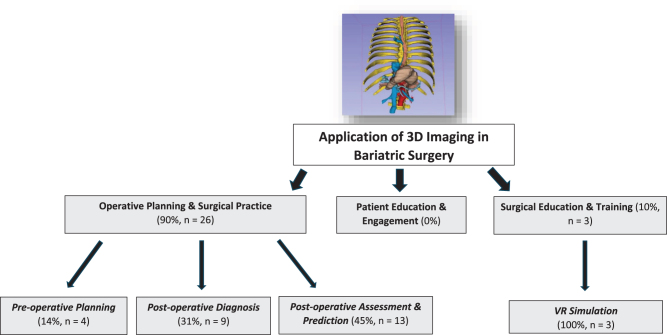
Application of 3D modeling in metabolic surgery.

The vast majority of included work focused on operative planning and surgical practice (90%, *n* = 26) (Fig. 2). This could be divided further into preoperative planning (14%, *n* = 4), postoperative diagnosis (31%, *n* = 9), and postoperative assessment and prediction (45%, *n* = 13). A small selection of work focused on surgical education and training (10%, *n* = 3). None of included literature studied the application of 3D modeling for patient engagement and education.

### Results of individual studies – narrative summary

#### Operative planning and surgical practice

Four papers utilized 3D modeling for preoperative guidance. Each describes creating virtual reconstructions of patient gastric anatomy, either through CT or MRI scans, to personalize metabolic operative procedures^[^25–27^]^. This is suitable for surgically naïve patients^[^25,26^]^ or complex revisional cases^[^27^]^.

To overcome the complexities of diagnosing postoperative bariatric surgical complications, nine papers demonstrated the benefit of 3D modeling^[^28–36^]^. For example, postoperative intra-thoracic migration is believed to be significantly underreported^[^54^]^. Numerous included studies have demonstrated 3D modeling is a superior modality for detecting migration, in both LSG and RYGB, compared to traditional methods^[^28,29,31,34,35,48,54^]^. Similarly, innovative applications of 3D modeling are shown to be effective for the diagnosis of sleeve dilation^[^31,36^]^ and reflux^[^30^]^. For one of the most feared bariatric complications, internal herniation^[^30^]^, 3D reconstructions of CT angiography were to shown to have potential improved diagnostic sensitivity^[^33^]^.

The remaining literature on operative planning described the postoperative assessment of GI anatomy and its correlation to metabolic clinical outcomes^[^37–44,47,49–51^]^. For LSG, a series of papers studied alternate gastric volumetric measurements and their impact on postoperative weight loss^[^38–41,43,44,47,49,50^]^. Almost all included papers reported 3D modeling and gastric volumetry as an accurate technique capable of predicting postoperative weight loss, however the determining measurements differed between papers. For instance, Lin *et al* highlighted the importance of gastric wall volume^[^43^]^, Hansen *et al* found total gastric sleeve volume of greater than 100 mL was a key indicator of poor TWL^[^40^]^ and Pawanindra *et al* argued the volume of resected stomach was the most important determinant of weight loss^[^44^]^. In contrast to other groups, Wickremasinghe *et al* found gastric volumetry was a poor predictor of postoperative weight loss and instead advocate for the utilization of gastric emptying half-time measured with nuclear scintigraphy.

Literature studying the volumetry of RYGB postoperative pouch anatomy had contrasting findings^[^37,42,52^]^. While all agreed on the accuracy of 3D pouch volumetry, Robert *et al* found no correlation between pouch size and postoperative weight lost, however, Ricciopo *et al* believe a small pouch is associated with faster emptying, better food tolerance and greater weight loss^[^37,42^]^. In a landmark paper by Ayuso *et al*, 3D pouch volumetry was utilized to investigate the contribution of pouch size to marginal ulcer (MU) formation. They found larger gastric pouches were prone to MU formation, with a 2.4-fold increase in MU risk for every 5 cm^3^. A lack of standardized measurements and outcomes assessments, in both the LSG and RYGB research, prevents direct comparisons.

#### Surgical education and training

A small group of studies described the contribution 3D modeling can make to bariatric surgical training^[^45,46,53^]^. These papers highlight the proficiency of VR training as a training technique for focused procedural development, such as single-port LSG^[^53^]^. Beyond single techniques, VR training was suggested as having a future central role in training programs and bariatric certification^[^45,46^]^.

### Results of syntheses – meta-analyses on 3D volumetry and abdominal circumference

Due to significant heterogeneity in outcome measures, it was not feasible to pool findings from identified literature on clinical outcomes. However, where possible, volumetric findings and abdominal circumference were pooled to understand the degree of consistency of 3D volumetry across all included studies. This would test the accuracy and reliability of 3D modeling.

**Figure 3.**
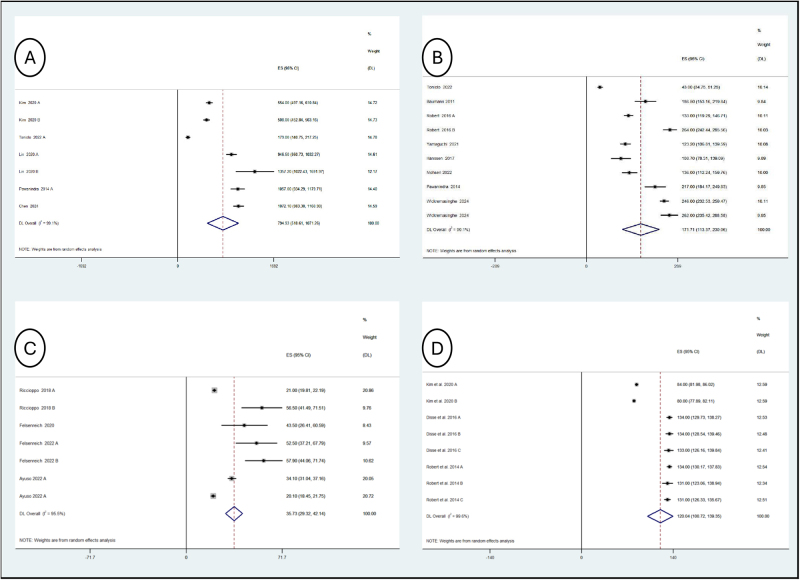
Results of volumetric synthesis. (A) Forest plot of preoperative gastric volume. (B) Forest plot of plosoperative gastric sleeve volume. (C) Forest plot of postoperative pouch volume. (D) Forest plot of abdominal circumference.

Five studies assessed preoperative stomach volume (*n* = 238) (Fig. 3A). Pooled analysis demonstrated an average preoperative stomach volume of 794.93 mL (95% confidence interval [CI]: 518.61–1071.26 mL; *I*^2^ = 99.1%). This is in keeping with existing literature on gastric volumes and existing research demonstrating obese individuals have the same stomach volume as those with a lower body mass index^[^55^]^.

For postoperative LSG gastric volume, eight papers were pooled (*n* = 328) (Fig. 3B). This found the average postoperative sleeve volume of 171.71 mL (95% CI: 113.37–288.58 mL; *I*^2^ = 99.1%). This matches existing literature using intraoperative fluid measurements for sleeve gastric volume^[^56^]^. The relatively wide (CI and substantial heterogeneity likely represent differences in operative technique, patient characteristics, and volumetric methodology.

Just four studies, containing a combined 251 individuals, measured postoperative pouch volume following RYGB & OAGB (Fig. 3C). On pooled analysis, the average pouch volume was 35.73 mL (95% CI: 29.32–42.14 mL; *I*^2^ = 95.5%). Once again, this closely mirrors pouch volumes measured using more rudimentary techniques^[^57,58^]^. Similarly to the gastric sleeve meta-analysis, significant heterogeneity is noted.

Finally, abdominal circumference was measured in 3 identified papers (*n* = 342) (Fig. 3D). On pooled analysis, the average circumference was 120.04 cm (95% CI: 100.72–139.35 cm; *I*^2^ = 99.6%). This is well above the recognized cut-offs for abdominal obesity^[^59^]^, which is in accordance with the expected abdominal circumferences of the observed preoperative bariatric population.

### Risk of bias

The NOS was utilized to grade included literature (Table 2). Overall, there was deemed a moderate risk of bias with an average score of 5.41. Upon categorization, 23 papers (79%) were considered moderate quality, with 4 (14%) scored as poor quality and 2 as high quality (7%).

**Table 2:** Newcastle–Ottawa Scale

Author, year	Selection	Comparability	Outcome	Overall score
Kim *et al* 2020	**	*	*	4
Toniolo *et al* 2022	**	*	***	6
Debs *et al* 2020	**		***	5
Felsenreich *et al* 2023	***		***	6
Sabry *et al* 2022	***		*	4
Arnoldner *et al* 2020	***	*	***	7
Elredge *et al* 2020	**	*	***	6
Baumann *et al* 2011	**		***	5
Wickremasinghe *et al* 2024	***	*	***	7
Chen *et al* 2024	***		***	6
Sahin *et al* 2023	***		***	6
Riccioppo *et al* 2018	***		***	6
Robert *et al* 2016	***	*	***	7
Blanchet *et al* 2010	***		**	5
Yamaguchi *et al* 2021	***		**	5
Klop *et al* 2018	**		**	4
Hanssen *et al* 2017	***		***	6
Mohsen Abd-Elfattah Moursi *et al* 2022	***		***	6
Alva *et al* 2008	*		**	3
Robert *et al* 2014	***		***	6
Karila-Cohen *et al* 2022	**	*	***	6
M. Felsenreich *et al* 2020	***		***	6
Lin *et al* 2020 A	***	*	***	7
Pawanindra *et al* 2014	***	*	***	6
Ayuso *et al* 2022	**	*	***	6
Disse *et al* 2016	***		**	5
Lewis *et al* 2012	***	*	*	5
Giannotii *et al* 2023 A	*		*	2
Barre *et al* 2019	**		**	4

#### Certainty of evidence

Certainty of evidence was graded low. Only observation studies were included, and the risk of bias was considered moderate on NOS. Evidence was limited by significant heterogeneity on *I*^2^ analysis. CIs on meta-analysis were consistent with existing literature, suggested relatively high precision, and outcomes were directly relevant to the population of interest. With studies reporting both positive and negative findings in relation to 3D modeling and a lack of industry funded studies, publication bias was deemed undetected^[^60^]^.

## Discussion

The authors outline the first systematic review aiming to understand the utilization of 3D modeling in metabolic surgery and performed meta-analyses on available volumetry outcomes and abdominal circumference measurements. As a new innovation, it is unsurprisingly the certainty of evidence is low, with moderate risk of bias. However, the review does highlight the potential impact of this emerging technology through its broad usage profile.

### Accuracy and potential roles of 3D volumetry in metabolic surgery

The review demonstrates the accuracy of 3D modeling for volumetric assessment of postoperative anatomy. For LSG, our meta-analyses demonstrated the average preoperatively gastric volume of 794.93 mL was reduced to 171.71 mL. For RYGB and OAGB, only postoperative pouch data was available, and the average volume was 35.73 mL. When measured, the average abdominal circumference was 120.04 cm. Each of these figures is in keeping with existing literature and therefore reflects the accuracy of 3D volumetry. Notable heterogeneity is attributable to variable patient characteristics, alternative operative techniques, and different modeling methodologies. Consequentially, this review was unable to correlate clinical outcomes with volumetric assessments due to significant heterogeneity in outcome measures. This highlights the necessity for standardized reporting structures^[^61^]^.

Considering its accuracy, 3D volumetry may be crucial for answering topical debates within metabolic surgery. For example, the optimal RYGB pouch size is still uncertain^[^62^]^. Arguably, a smaller remnant stomach will achieve earlier satiety, produce less acid and reflux symptoms, and may have greater clinical outcomes as a result. However, a relatively larger pouch will allow lower intra-gastric pressure therefore possibly reducing reflux complications and improve patient tolerance^[^63^]^. Pouch shape is another critical consideration. Traditionally, a long and narrow pouch was believed to be preferable. However, recent evidence included in this review highlights a shorter and broader pouch could lead to greater weight loss, reduced GORD and a lower prevalence of MU^[^51^]^. Additionally, the shape and volume of a sleeve or pouch could have profound effects on postoperative nausea, vomiting, and reflux symptoms. A well-constructed RCT incorporating 3D modeling and volumetrics may satisfyingly answer these key surgical questions.

### 3D modeling for in preoperative planning and postoperative assessment

The review uncovered early evidence exploring the potential benefit of 3D modeling for preoperative guidance in metabolic surgery. However, the technique is underutilized in comparison to other surgical specialties, such as orthopedics, which have already established the feasibility of 3D bioprinting to improve operative planning^[^64^]^. There is clear value in 3D reconstructions, especially for complex revisional bariatric surgery, where a deep understanding of the patient’s anatomy will allow for superior operative planning^[^65^]^. Additionally, as highlighted by Toniolo *et al*^[^66^]^, 3D modeling permits intricate biomechanical assessment of a patient’s gastric anatomy. This provides an opportunity for greater personalized care and targeted patient selection for innovative minimally invasive techniques, such as Endoscopic Sleeve Gastroplasty^[^67^]^.

A notable finding was the feasibility of using 3D modeling to measure abdominal circumference. This could have significant benefit. For example, as abdominal circumference has been shown to be a suitable surrogate abdominal obesity and related mortality^[^68^]^, there may be an opportunity to use 3D modeled abdominal circumference cutoffs to select and plan bariatric operative cases. Furthermore, abdominal circumference may be a valuable predictor of patients with high laparoscopic operative torque. In the age of robotic surgery, this may help select those patients that would benefit greatest from a robotic approach^[^69^]^. Additionally, improved accuracy for simulation would advance related technologies including Artificial Intelligence (AI) intraoperative guidance and next generation device development. AI-powered algorithms can enhance the interpretation of 3D models by identifying critical anatomical structures, predicting surgical outcomes, and simulating operative steps in real time. This fusion could enable highly personalized surgical plans, optimized patient outcomes, and refined surgical techniques. AI-driven tools can also improve patient selection for novel procedures by using predictive analytics based on biomechanical data from 3D reconstructions, further improving the precision of metabolic surgery^[^70^]^.

Postoperative bariatric complications are notoriously challenging to diagnose^[^71^]^. Therefore, it is highly reassuring that 3D modeling has been shown to provide diagnostic benefit. When combined with AI, such diagnostic capabilities could be further enhanced, allowing automated identification of postoperative complications, reducing diagnostic delays, and improving clinical outcomes. Beyond helping patients avoid unnecessary surgical procedures, this approach could provide logistical and financial benefits for hospitals. However, a large-scale RCT is required to confirm the advantage of 3D modeling in the diagnostic setting. Researchers should also consider the added value of AI-enhanced 3D models, which will strengthen the case for their clinical application by providing surgeons with highly detailed, actionable outputs.

### *3D* modeling *for clinician and patient education*

As medicine modernizes, the traditional surgical apprenticeship model must evolve and incorporate innovative educational technologies^[^72^]^. Competency-based training structures employing cheaper and more ethically acceptable replacements to cadaveric or animal simulators, such as VR, AR, or 3DP models, are emerging as attractive alternatives for both trainees and trainers^[^73^]^. The literature identified in this review demonstrates metabolic surgery is capitalizing on these innovations and is making early advances to imbed VR into training schemes. In other surgical specialties, existing research details the benefits of using 3D reconstructions to individually assess trainees and help tailor training programs^[^13^]^. Researchers in metabolic surgery may well consider using similar techniques or investigate other 3D modeling adjuncts such as AR and their impact on the quality of surgical training^[^74^]^. Outside simulation, it is worth highlighting preoperative 3D reconstructions have substantial merits for improving trainees’ intraoperative performance.

It is well established that 3D models, either virtual or physical, provide significant value for patient education^[^14,75^]^. In metabolic surgery, effective education is essential and has been linked to improved postoperative outcomes^[^76^]^. Models act as a powerful tool in the consenting process and assist patients in understanding the intricate steps of a bariatric procedure. Therefore, the lack of research into personalized 3D models in metabolic surgery could be considered a missed opportunity. This would be a valuable avenue for future research. Personalized 3D models could be a risk-free intervention that may improve both the patient experience and their quality of life.

### Future trends and considerations

All 3D reconstructions within the included studies were virtual models. Metabolic surgery appears to be behind other surgical specialties, who have utilized variations of 3D modeling such as intra-operative AI guidance to identify critical structures^[^77–79^]^ or 3DP models for realistic surgical training^[^80^]^. Crucially these techniques integrate well with robotic surgery^[^81^]^. For metabolic surgeons, combining robotics with 3D reconstructions could allow for picture-in-picture guidance during complex revisional work, outline key anatomical structures or automatically measure the limb length during RYGB. As tissue is manipulated during surgery, AI could update 3D reconstructions to reflect real-time anatomical changes, enhancing decision-making. Furthermore, these 3D models could predict potential complications or guide optimal suture placement, appropriate direction of dissection thin tissue planes based on the patient’s unique anatomy. Therefore, to allow bariatric surgeons to maximize the benefit of 3D modeling several steps are required. This includes large-scale validation studies to assess the impact of integrating 3D reconstructions with robotic platforms on surgical outcomes, operative efficiency, and training effectiveness.

Perhaps, we are on the cusp of widespread adoption of 3D modeling technology. In Western countries, numerous studies have outlined the growing cost effectiveness of 3D technologies^[^82,83^]^ and its increasing prevalence in healthcare settings^[^84,85^]^. This trend is repeating across the world^[^86,87^]^, especially with both commercial^[^88^]^ and open-access 3D modeling platforms available^[^89^]^. Therefore, given the rising global trend and enhanced accessibility of 3D modeling, metabolic surgery should comprehensively outline all suitable clinical applications of this technology.

### Strengths and limitations

There are numerous strengths to this paper. For example, this is the first systematic review assessing the utilization and benefit of 3D modeling in metabolic surgery. A publicly registered protocol ensures the review was performed to accepted standards. The combination of a narrative review with meta-analyses of available data allowed the authors to assess disparate clinical outcomes and provide a broad overview of current 3D modeling practices. The review was limited by significant heterogeneity in clinical data, low certainty of evidence in included studies and is restricted to publications in English. The authors actively chose not to exclude papers based on quality to ensure a wide capture of publications, however this may lead to overrepresentation of low-quality work.

## Conclusions

In conclusion, this is the first systematic review on the utilization of 3D modeling in metabolic surgery. The review highlighted the accuracy of 3D modeling for volumetric assessments and its developing role in surgical planning and practice. Bariatric surgery has made significant advances integrating VR into surgical training. However, there are numerous opportunities to further evaluate the role of 3D modeling in metabolic surgery. This may well include the use of AR or 3DP models, the benefit of 3D reconstruction for patient education and using 3D volumetric assessments to answer fundamental clinical questions. Ultimately, the fusion of 3D modeling, AI, and robotics could redefine the standard of care in metabolic surgery, pushing the boundaries of precision, safety, and innovation.

## Data Availability

Not applicable.
